# Safety of Epidural Hyaluronic Acid Injections in Managing the Symptoms of Lumbar Foraminal Stenosis: A Prospective Preliminary Study

**DOI:** 10.3390/jcm12062359

**Published:** 2023-03-18

**Authors:** Piotr Godek, Kuba Ptaszkowski

**Affiliations:** 1 Sutherland Medical Center, 04-036 Warsaw, Poland; 2 Department of Physiotherapy, Wroclaw Medical University, 50-368 Wrocław, Poland

**Keywords:** lumbar foraminal stenosis, radicular pain, hyaluronic acid, epidural injections, nerve root neurodynamics, neuromobilization

## Abstract

Lumbar foraminal stenosis (LFS) of degenerative origin is a common reason for distorted neurodynamics of nerve roots, causing radicular pain that is difficult to resolve with conservative treatments. A hyaluronic acid (HA), providing a sliding layer in the mechanical interface of a nerve root in a narrowed lateral recess, could potentially improve its neurodynamics and the trophic, leading to radicular pain reduction and improvement of function. This study aimed to assess the usefulness of ultrasound-guided HA epidural injections combined with neuromobilization in the conservative treatment of LFS. A group of 10 consecutively admitted patients with MRI-confirmed LFS and reduced straight leg raise (SLR) test results were qualified for a single HA epidural injection along with self-performed neuromobilization. Three measurement tools were used for primary outcomes: the numeric rating scale (NRS) for pain intensity, the Oswestry disability index (ODI) and the Roland–Morris questionnaire (RMQ) for disability level, and the angle of pain-free elevation in the SLR test as a functional assessment. The treatment was accomplished in all patients (100%). Overall, 60% of the patients completed all follow-up visits. There were no statistically significant differences regarding the results of the NRS, ODI, or RMQ; however, a statistically significant increase in the results of the SLR test was noted (*p* = 0.015). Three patients reported a flare-up of the symptoms shortly after injection but without neurological deficits. In conclusion, an epidural HA injection combined with a self-administered exercise program is a promising method and might be a beneficial way to enhance the neurodynamics of nerve roots in LFS and offer an option for steroid treatment. However, this method of epidural HA administration in LFS should be verified in further studies to confirm its efficiency and safety.

## 1. Introduction

Although low back pain (LBP) pathogenesis is very heterogenic, intervertebral disc degeneration (IVD) is commonly regarded as a cornerstone of the cascade of phenomena that produce lumbar degenerative spondylosis (LDS). It is estimated that the majority of the population is supposed to experience LBP at some point in their lives. The 1-year incidence of the first-ever LBP episode varies from 6.3 to 15.4%, while the 1-year incidence of any LBP episode varies from 1.5 to 36%, with episode remission at 1 year ranging from 54 to 90% [1].

Lumbar foraminal stenosis (LFS) is one of the most frequent forms of LDS, a late consequence of IVD and considered a wear-and-tear phenomenon that is at least partially age-related. The narrowing of the intervertebral space leads to secondary hypertrophy of the intervertebral joints as a reaction to overload, ligamentous overgrowth, and a reduction in intervertebral foramen diameter. This chain reaction is the main reason for nerve root impingement and distorted neurodynamics that lead to mixed pain in advanced spondylosis (axial and radicular pain) [2]. Nerve root compression comes not only from a purely mechanical limitation of space.

Another, no less important, cause of nerve root impingement and distorted neurodynamics is adhesion (epidural fibrosis) formation in the border zone between the nerve sheaths and the periosteum of the bone frame in the course of inflammation that accompanies every degenerative process [3]. This may explain the often-observed discrepancy in clinical practice between the intensity of degenerative and the proliferative changes observed in the MRI scan and the intensity of the pain (e.g., the opposite side of the symptoms to the dominant level of LFS).

Conservative treatment of LFS with radicular symptoms is very difficult. The mainstream of care is steroid injections with a rehabilitation program that particularly emphasizes manual therapy, including specific neuromobilization techniques [4,5]. Nevertheless, manual therapy techniques used to improve the neurodynamics of the nerve root in the presence of adhesions are sometimes ineffective because the nerve mobilized in the peripheral part to the entrapment site has been stretched [6,7]. In that case, the border zone adhesions persistently reduce the blood supply and nourishment of the nerve at the entrapment site as the physical therapist more ambitiously tries to release the nerve. This often causes frustrating worsening of symptoms and unpleasant post-treatment flare-ups, despite the efforts of the physiotherapist and the patient [6].

One of the methods of preventing adhesions in the border zone is using HA or interposition materials in spine surgery; HA gel has also been described in experimental animal studies [7]. HA injections have been used as a treatment for lumbar pain, with some studies reporting positive results, especially in the course of intervertebral disc degeneration [8]. Further, epidural administration of HA under ultrasound guidance followed by neuromobilization may hypothetically improve the sliding capabilities of the nerve sheaths in the tight space of the lateral recess and the intervertebral foramen, contributing to the improvement of the nerve root tropics and, consequently, reducing radicular pain.

There is also an anti-inflammatory effect of HA, which is a naturally occurring substance, particularly found in joint fluids and connective tissues [9]. HA has anti-inflammatory properties, which makes it a promising treatment option for various inflammatory conditions [10]. HA injections are commonly used to treat joint pain and osteoarthritis, in which inflammation is a contributing factor to pain and degeneration [11,12]. HA works by binding to receptors on immune cells, reducing their activity and preventing the release of inflammatory cytokines [13]. HA has been shown to have anti-inflammatory effects by reducing the expression of genes for proinflammatory cytokines (IL-1, IL-6, and IL-8; TGF-beta and TNF-alpha) [14] and reducing the production of prostaglandins and metalloproteinases (MMP-1 and MMP-13; ADAM-TS4 and ADAM-TS45; or TIMP1) [15]. Although the exact mechanism of action is still being studied, the anti-inflammatory effect of hyaluronic acid injections is believed to contribute to their therapeutic benefits [16].

To date, only one case report has been reported in which the combined interposition treatment with HA administration to the root recess was successfully applied using the epidural method under ultrasound guidance with a rehabilitation program based on neuromobilization techniques [17]. Therefore, the present study is a continuation of research on this subject and, as a pilot study, is designed to evaluate the tolerance and safety of epidural HA injection and neuromobilization in the symptomatic treatment of LFS. The principal hypothesis is that epidural injection of HA with additional neuromobilization reduces radicular pain and improves function in patients with LFS.

## 2. Materials and Methods

### 2.1. Study Design and Participants

This open-label, prospective, non-randomized, controlled, and interventional clinical trial was conducted at Sutherland Medical Center (SMC) in Warsaw, Poland. A final group of 10 patients participated in this research project. Patients who successfully completed the qualification were enrolled sequentially in order of admission to the SMC clinic. The median duration of LFS symptoms was 9 weeks.

### 2.2. Ethical Considerations

The trial protocol was accepted by the Institutional Review Board at the Wroclaw Medical University (approval no. KB-344/2022, approval date: 27 April 2022). The entire research project was completed in accordance with the Declaration of Helsinki and Good Clinical Practice standards. In addition, the written informed consent was obtained from all patients before the study enrollment. The study was prospectively registered on the ISRCTN registry platform (identification number: ISRCTN14155217, registration date: 16 May 2022; accessed on 16 May 2022 https://doi.org/10.1186/ISRCTN14155217; trial acronym HALFS: Hyaluronic Acid in Lumbar Foraminal Stenosis).

### 2.3. Qualification Criteria

Inclusion criteria comprised: (1) clinical symptoms of radicular pain and nerve root impingement in the lumbar region, (2) MRI-confirmed LFS of degenerative origin, (3) patients’ consent for epidural HA injection, and (4) the appropriate level of cooperation ensuring proper implementation of neuromobilization. Exclusion criteria were: (1) other spine diseases causing LFS (post-traumatic, systemic inflammatory diseases, cancer, congenital defects), (2) neurologic deficits, (3) current or previous spinal injuries requiring different treatment options, (4) the presence of contraindications for anticoagulants and other injections, and (5) impeded contact with the patient or not promising cooperation during implementation of neuromobilization.

### 2.4. Recruitment Procedure

**Figure 1:**
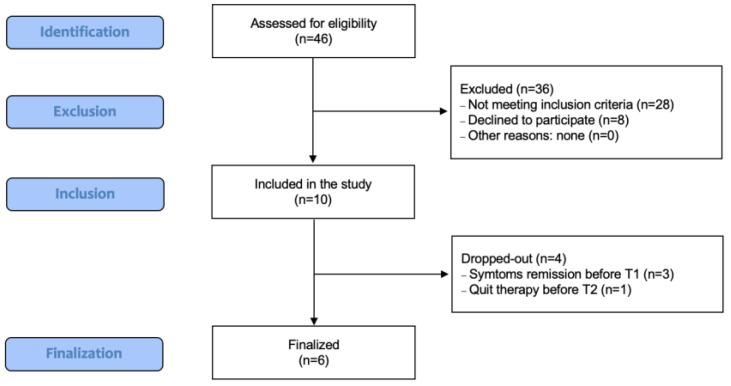
Flow chart of the study participants.

A group of 46 subjects was assessed for eligibility, of which 10 had no exclusion criteria and were qualified to participate in the study from 10 June 2022 to 9 September 2022. The recruitment start date was 1 June 2022. Four out of ten patients did not complete the trial program. Three patients had flare-ups of the symptoms shortly after HA administration and did not even have a follow-up control visit. One patient quit observation after the second monthly follow-up and asked for another treatment because of no improvement. Figure 1 presents a flow chart of the participants in the subsequent study stages.

### 2.5. Study Outcomes

There were four primary outcomes in this study: pain intensity was assessed using the numeric rating scale (NRS) score from 0 to 10, where 0 indicates no pain and 10 is maximal pain), the Oswestry disability index (ODI) from 0 to 50, where 0 indicates no disability and 50 is maximal disability), the Roland–Morris questionnaire (RMQ) from 0 to 24, where 0 indicates no disability and 24 is maximal disability), and the straight leg raise (SLR) test performed passively, with the outcome registered in degrees measured by an inclinometer. Follow-up observation for primary outcomes included initial assessment (IA), as well as first (T1), second (T2), and third (T3) monthly assessments following intervention. The secondary outcome was to assess possible adverse events and reasons for withdrawal from the study at three time points (T1, T2, and T3). Ultrasound spinal examination was conducted with the use of an Alpinion E-CUBE 12 device (Alpinion Medical Systems Co., Ltd., Seoul, Republic of Korea), convex transducer SC1-4H (1–4 MHz). The interlaminar space for epidural access was identified in the parasagittal plane over the LFS level.

### 2.6. Treatment Protocol

Participants were enrolled consecutively in the study. Before the injection, the patient received instruction on how to perform the neuromobilization of nerve roots in a lateral decubitus position with the use of the skateboard. It was emphasized that the most important element was not to reach beyond the pain-free angle of swinging, using gentle and very slow movement without any tension in the lumbar region. The injection was performed in the flexion prone position of the patient under local anesthesia. An epidural ultrasound-guided injection with interlaminar access was chosen. After the identification of epidural space, the needle BD 20GA 3.5″ (0.9 × 90 mm) was introduced in-plane until it reached the ligamentum flavum. Then, using the loss of pressure technique, the epidural space was identified by administering 5 mL of saline. If proper spreading was achieved, the volume of 2 mL of HA (Sinovial HL, IBSA) was slowly injected with the constant observation of active ipsilateral foot movement. The Sinovial HL product was chosen because of its small volume of injectate; it has also been registered for extra-articular administration (tendons and ligaments) [18]. Each patient was observed 30 min after injection and the exercise program was launched the following day.

### 2.7. Statistical Analysis

The statistical analysis was performed using Statistica for Windows, Version 13 (TIBCO Inc., Palo Alto, CA, USA). The number (n) of subjects was indicated and the median (Me) and interquartile range (IQR) were calculated for the description of variables. In order to verify the normality of the distribution of the variables under study, the Shapiro–Wilk test was used. For qualitative variables, the frequency of their occurrence (percentage) was calculated. Comparison of results between successive measurements was carried out using Friedman’s rank test. The statistical significance level was set at *p* < 0.05.

## 3. Results

The treatment was accomplished in all patients (100%). All check-up visits were passed by 60% of the patients. A detailed demographic characteristic of the study participants is shown in Table 1.

**Figure 2:**
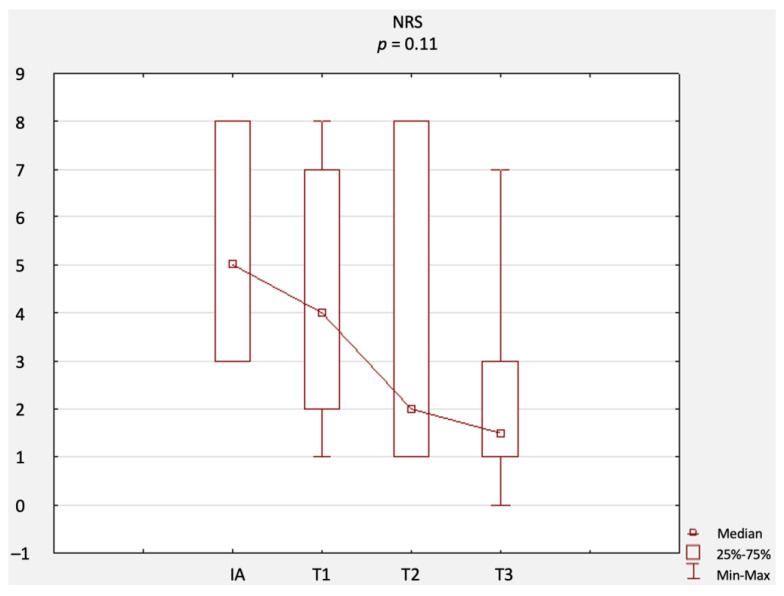
Comparison of NRS results at IA, T1, T2, and T3. Abbreviations: NRS, numeric rating scale; IA, initial assessment; T1, first-month assessment; T2, second-month assessment; T3, third-month assessment.

**Figure 3:**
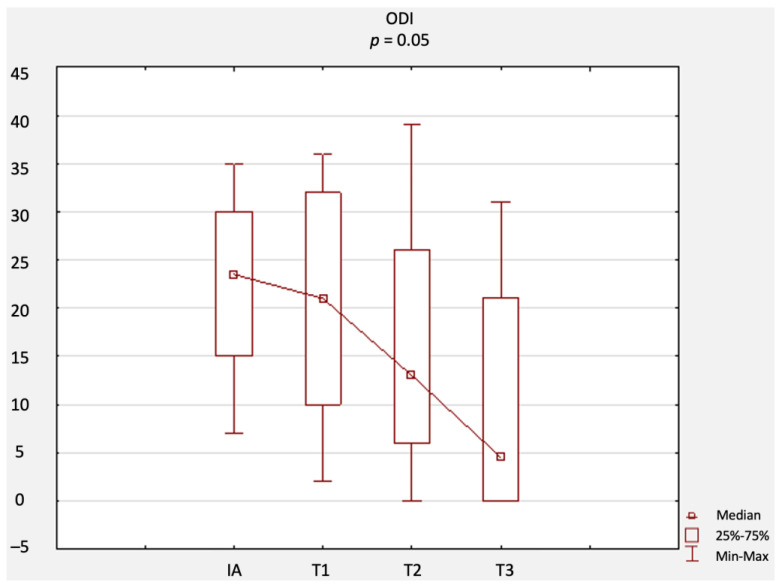
Comparison of ODI questionnaire results at IA, T1, T2, and T3. Abbreviations: ODI, Oswestry disability index; IA, initial assessment; T1, first-month assessment; T2, second-month assessment; T3, third-month assessment.

**Figure 4:**
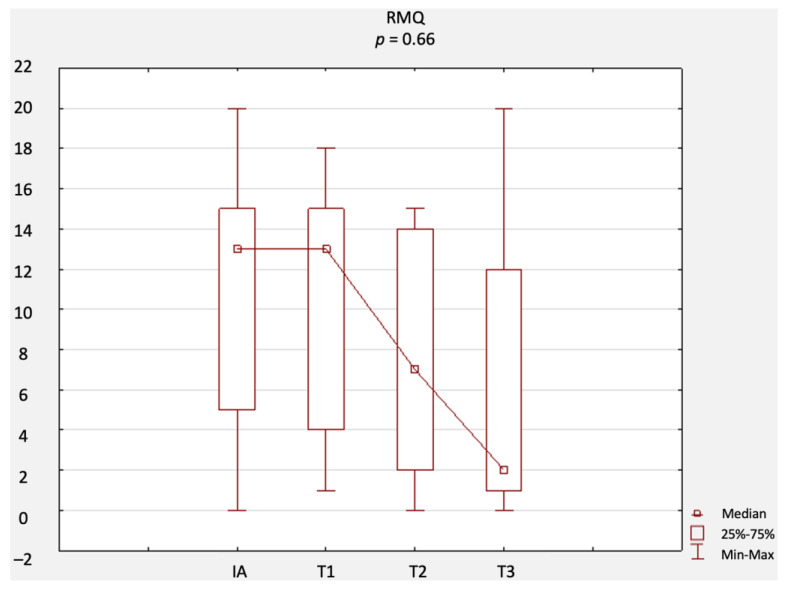
Comparison of RMQ results at IA, T1, T2, and T3. Abbreviations: RMQ, Roland–Morris questionnaire; IA, initial assessment; T1, first-month assessment; T2, second-month assessment; T3, third-month assessment.

**Figure 5:**
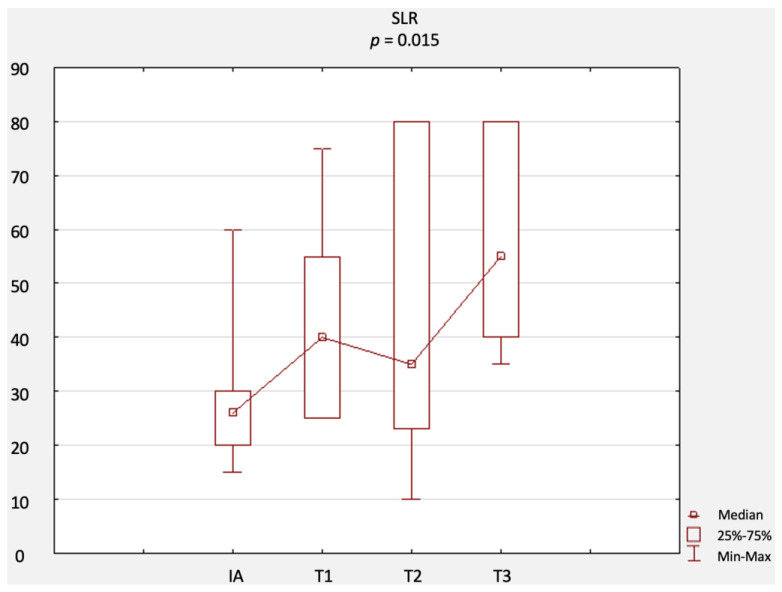
Comparison of SLR results at IA, T1, T2, and T3. Abbreviations: SLR, straight leg raise; IA, initial assessment; T1, first-month assessment; T2, second-month assessment; T3, third-month assessment.

A comparison of changes in scores related to primary outcomes (including results of the NRS, ODI questionnaire, RMQ, and SLR test) is provided in Figure 2, Figure 3, Figure 4 and Figure 5. There were no statistically significant differences in terms of the results of the NRS (Figure 2), ODI (Figure 3), or RMQ (Figure 4). However, a statistically significant increase in the results of the SLR test was noted (Figure 5).

Three patients shortly after injection experienced a flare-up of the symptoms but without neurological deficit. One patient improved at T1 control, but, at T2, they decided to change therapy because of intensive pain.

## 4. Discussion

HA injections are a popular symptomatic management method applied to patients with osteoarthritis (OA) and there is strong evidence confirming that HA could increase quality of life in cases of mild to moderate OA [19]. Performing extra-articular HA injections mainly concerns tendons and is aimed at preventing inflammatory adhesions. The off-label use of HA also includes many FDA-approved indications in the fields of aesthetic medicine for tendons, ligaments, and even muscles [20].

It is worth noting that HA has long been used as a preventive method for post-surgical adhesions in the cosmetic surgery area. This indication was demonstrated by Wang et al. [21], who applied epidural cross-linked HA gel in a preclinical study. This study revealed the modification of scar gene expression and proved the prevention of epidural adhesion after laminectomy. Based on a histomorphometry examination at 2 weeks following surgery, the number of fibroblasts in the study group showed a significantly lower number of fibroblasts compared with the control group and the CT scans revealed that the cross-linked HA gel effectively reduced the epidural space adhesion after the laminectomy procedure [21]. Analogous findings were reported by Iik et al. [7] in preclinical trials using cross-linked high-molecular-weight HA administered to rats undergoing neurosurgical procedures of laminectomy and discectomy. A significantly lower fibroblast cell density, tissue hydroxyproline concentration, dural adhesion, foreign body response, granulation tissue, and epidural fibrosis were found compared with the control group [7].

HA also exerts immunosuppressive and cytoprotective effects, represented by increased levels of intracellular storage of ATP, increased integrity of DNA, promoted anti-inflammatory effect and decreased concentrations of pro-inflammatory cytokines (IL-G), and reduced prostaglandins and metalloproteinase production through the CD44 receptor [22,23]. The molecule also has the capability of self-replication as it stimulates endogenous production in the joint where it is administered. It also exhibits analgesic effects by lowering the excitability of free nerve endings through the nociceptive inhibition of substance P and decreasing PGE2 and bradykinin as mediators of hyperalgesia while lowering the excitability threshold of opioid receptors [24].

To the authors’ knowledge, no current research has assessed the safety and efficacy of epidural HA injections in human subjects. The only case study published by the author in 2022 on the symptomatic treatment of LFS with radicular pain was the inspiration for launching the pilot study [6]. The idea was to check whether creating an interposition slip layer for the nerve root sheaths in a tight lateral recess may improve the progress of its neurodynamics and, by regaining its natural mobility, ensure a normalized trophic condition and absorb inflammatory-related edema.

The application of Sinovial HL 3.2% (IBSA) with a hybrid composition of double-weight molecules was selected for the therapy for several reasons. First, it is a highly purified product obtained through a fermentation process and does not cause allergic reactions in animals. Second, 2 mL of the administered agent constitutes a small volume for the therapy. Third, registration of the product allows for its extra-articular administration. Fourth, the double size of particles (HMW has very good rheological properties due to LMW, which triggers endogenous HA production by stimulation of fibroblasts). Lastly, participants in previous studies showed very good tolerance of the product administered together with PRP and no cytotoxic effect [25,26,27,28].

The results obtained are inconsistent, with both very satisfactory and stable results observed, as well as unsatisfactory findings requiring changes in therapy due to increased pain intensity. It is unclear why some patients experienced a gradual and lasting improvement and others deteriorated shortly after injection. However, they did not report any significant pain, apart from tension during the injection or during the 30-min observation in the clinic. The reason may be the tightness of the lateral recess in the vertebral canal and the perineural pressure prevailing there, which cannot be accurately assessed, even with a high-resolution MRI. Perhaps the difference in outcome is related to the irregular distribution of the HA around the nerve in the presence of adhesions, because then it is possible that the drug accumulates in the lacunas rather than along the nerve sheath like a lubricated string on a pulley, as desired for effective neuromobilization. Another reason may be the incorrect methodology of exercising by the patient, despite seemingly very detailed instructions. Unfortunately, during subsequent control visits, the exercise methodology often differed significantly from the recommended pattern, which can be considered a study limitation. However, our goal was to avoid human factors that could introduce bias if neuromobilization was performed through manual therapy.

Part of the solution to the problem of irregular drug distribution could be injections under the control of CT with direct observation of HA spreading and exercise performance could be coupled with some type of biofeedback that would at least correct the starting position for the exercise. This study should be continued considering the above issues, improving the methodology, and eliminating the limitations. Other questions that need elucidation are: (1) Is a single HA injection sufficient for fibrous scar tissue resolution and to secure proper and stable surroundings for impinged nerve roots before the process of neuromobilization? (2) How many doses and in what schedule should it be offered? (3) Should dosing be preceded by spinal traction to secure more space? Further investigations addressing these questions should be conducted.

In summary, while the volume of the injected agent is an important factor to consider, it is not the only factor that determines the success of the treatment in pain reduction and functional improvement. Other factors, such as the patient’s individual response to the treatment, the severity and duration of the condition, the accuracy of the injection technique, and the patient’s adherence to recommended exercise and rehabilitation protocols, can all have an impact on the outcome of the treatment. Furthermore, it is important to note that the effectiveness of HA injection therapy should not be evaluated in isolation but in comparison with other forms of injection and conservative treatments. Research has shown that HA injections can be effective in reducing pain and improving function in patients with various musculoskeletal conditions, such as osteoarthritis and tendinopathy, when administered properly and in appropriate doses [29,30]. However, the results are often mixed and depend on various factors, as mentioned above. In comparison with other injection therapies, such as corticosteroid injections, HA injections may provide a more sustained effect and have a lower risk of adverse effects, making them a viable option for some patients. The success of HA injection therapy in pain reduction and functional improvement cannot be solely attributed to the volume of the injected agent but rather a combination of various factors. Comparing the effectiveness of HA injections to other forms of injection and conservative treatments can provide a better understanding of their potential benefits and limitations.

### Strengths and Limitations

This is the first worldwide in vivo study to address the potential of HA epidural injections to improve nerve root sliding during neuromobilization. The trial confirmed the safety of the HA: no serious complications, such as neurological deficits, allergic reactions, or sudden intensification of symptoms, were noted during its administration or during the immediate observation period. However, there are several limitations to be discussed. Undoubtedly, despite the primary character of the study, its methodological limitations include a relatively small group of participants, operator-dependent shortcomings in ultrasound-guided epidural HA administration (impossible direct visualization of HA spreading), the lack of a control group or sample blinding, and bias related to the process of neuromobilization ordered for the patient.

Nevertheless, our study provides valuable insights into the practical results of HA epidural administration in clinical practice in LFS, which are highly relevant to the aims of this special issue. However, evaluating the safety of a medical procedure is an important aspect of clinical practice and can inform future research on its therapeutic effects. Thus, it is important to recognize that research on the safety of a medical procedure is an integral part of the research process and should not be dismissed as irrelevant. By evaluating the safety and feasibility of a treatment, researchers can inform and guide future research on its effectiveness and ultimately improve patient care, especially the safety and efficiency of using HA epidural administration in LFS. Without a doubt, a case series with a small number of patients may have limitations in terms of generalizability, however it can still provide valuable insights into the clinical effectiveness and safety of a treatment in a specific patient population. Preliminary studies are often used to generate hypotheses and identify potential areas for further investigation and can still make important contributions to medical knowledge and inform future research.

## 5. Conclusions

Epidural HA injection combined with a self-administered exercise program may be a beneficial way to enhance the neurodynamics of nerve roots in LFS and offer an option for steroid treatment. Our pilot study focused on evaluating tolerability and safety and indicated epidural HA injection as a promising treatment option for LFS, considered when conservative treatment fails. However, there is still a lack of strong evidence as there are not many studies, hence it is impossible to formulate strong conclusions and treatment recommendations at this stage.

Further investigations should objectively depict the distribution of the HA in the surrounding nerve root to find any correlation between the MRI image and the pattern of nerve lubrication and to identify the prognostic factors for this type of intervention. Subsequent studies should be performed on a larger group of patients using a double-blind technique and a control group. The neuromobilization methodology should be standardized to avoid imposing a bias on the role of HA alone. This method of HA administration in LFS should be used with caution, as three of the cases experienced a flare-up of the symptoms shortly after injection, although without a neurological deficit.

## Figures and Tables

**Table 1:** Characteristics of the study participants.

Study Group (n = 10)		Me	IRQ
Age (years)		52.5	25.0
Body height (cm)		173.5	10.0
Body weight (kg)		78.5	18.0
Duration of symptoms (weeks)		9.0	9.0
		n	%
Sex	Female	5	50.0
	Male	5	50.0
Disease phase	Chronic	5	50.0
	Acute	3	30.0
	Subacute	2	20.0
Side of pain	Left	7	70.0
	Right	3	30.0
Hernia form	Extrusion	4	40.0
	Rudimentary	1	10.0
	Sequestration	2	20.0
	Prolapse	3	30.0
Dominant level of hernia	L5/S1	7	70.0
	L2/L3	1	10.0
	L4/L5	2	20.0
A form of discopathy	One-level	3	30.0
	Multi-level	7	70.0
Diabetes (yes)		0	100.0
Peripheral vascular disease (yes)		1	10.0
Bone metabolism disorders (yes)		1	10.0
Polyneuropathy/myopathy (yes)		2	80.0
Psychosomatic (yes)		0	100.0
Spine surgeries (yes)		1	10.0

Abbreviations: Me, median; IRQ, interquartile range, n, number of participants.

## Data Availability

The data presented in this study are available on request from the corresponding author.
